# Trauma Center Staffing, Infrastructure, and Patient Characteristics that Influence Trauma Center Need

**DOI:** 10.5811/westjem.2014.10.22837

**Published:** 2014-11-11

**Authors:** Mark Faul, Scott M. Sasser, Julio Lairet, Nee-Kofi Mould-Millman, David Sugerman

**Affiliations:** * Centers for Disease Control and Prevention, Atlanta, Georgia; † Emory University, Department of Emergency Medicine, Atlanta Georgia; ‡ University of Colorado, Department of Emergency Medicine, Aurora, Colorado

## Abstract

**Introduction:**

The most effective use of trauma center resources helps reduce morbidity and mortality, while saving costs. Identifying critical infrastructure characteristics, patient characteristics and staffing components of a trauma center associated with the proportion of patients needing major trauma care will help planners create better systems for patient care.

**Methods:**

We used the 2009 National Trauma Data Bank-Research Dataset to determine the proportion of critically injured patients requiring the resources of a trauma center within each Level I–IV trauma center (n=443). The outcome variable was defined as the portion of treated patients who were critically injured. We defined the need for critical trauma resources and interventions (“trauma center need”) as death prior to hospital discharge, admission to the intensive care unit, or admission to the operating room from the emergency department as a result of acute traumatic injury. Generalized Linear Modeling (GLM) was used to determine how hospital infrastructure, staffing Levels, and patient characteristics contributed to trauma center need.

**Results:**

Nonprofit Level I and II trauma centers were significantly associated with higher levels of trauma center need. Trauma centers that had a higher percentage of transferred patients or a lower percentage of insured patients were associated with a higher proportion of trauma center need. Hospital infrastructure characteristics, such as bed capacity and intensive care unit capacity, were not associated with trauma center need. A GLM for Level III and IV trauma centers showed that the number of trauma surgeons on staff was associated with trauma center need.

**Conclusion:**

Because the proportion of trauma center need is predominantly influenced by hospital type, transfer frequency, and insurance status, it is important for administrators to consider patient population characteristics of the catchment area when planning the construction of new trauma centers or when coordinating care within state or regional trauma systems.

## INTRODUCTION

In the United States, unintentional injury is the leading cause of death for people aged 0–44 years of age.^1^ Treatment of severely injured persons at a Level I trauma center compared to a non-trauma center has been associated with a 25% reduction in mortality.^2^ Published guidance from the U.S. Centers for Disease Control and Prevention (CDC) provides detailed prehospital transport recommendations for trauma center destination for severely injured patients meeting specific criteria.^3^ A better understanding of how infrastructure, staffing and patient characteristics within a trauma center is impacted by the proportion of patients requiring advanced trauma care is critical for better trauma system management.

Given the complexity of traumatic injuries, trauma centers are, by design, large, resource-intensive environments, capable of providing patients with a wide array of trauma and non-trauma care services, including access to complex diagnostic equipment, intensive care unit (ICU) beds, and trauma care clinical expertise through varied medical and surgical specialists.^4^ These resources are readily available 24 hours a day, seven days a week. Although effective, the resources available at a trauma center can be costly. In one study, the cost of trauma center readiness (excluding trauma care costs) was $2.7 million annually,^5^ while another study reported the increased cost of treatment at a trauma center compared to a non-trauma center as being over $7,264 per patient.^6^

The American College of Surgeons (ACS) delineates 108 specific criteria for formal trauma center verification, including the volume of trauma patients seen, volume of inpatient trauma admissions, continuous availability of specialty staff, and provider-to-patient ratios for every trauma center.^7^ Trauma center verification criteria, in part, dictate resource allocation and use by trauma centers. However, the relationship between ACS trauma center verification criteria and patient use of those resources has not been fully explored.

In 2004, Laurent et al. studied and reported that higher trauma center patient volumes were not associated with improved patient outcomes,^8^ thereby refuting previous findings suggestive of a mortality benefit at trauma centers with higher patient volumes.^9^–^12^ Thus, the evidence of patient volume has been mixed. Part of the inconsistency may be due to differences in the proportion of patients that require the services of a trauma center. Additionally, trauma centers, like public safety agencies, require continuous staffing, regardless of patient volume, with an unclear cost-benefit relationship. In terms of nursing, higher numbers of more experienced nursing staff confer a survival benefit to severely injured patients receiving trauma care in trauma centers, as opposed to using less experienced nursing staff as a resource-conserving mechanism.^13^

The American College of Surgeons Committee on Trauma (ACS-COT) recommends neurosurgery, orthopedic and trauma specialist availability 24 hours a day for Level I and II trauma centers. The value of these clinical specialties’ involvement in trauma care has been well established.^14^ Continuous neurosurgical care, for example, is generally required to apply the Brain Trauma Foundation treatment guidelines. Yet, the literature has raised the issue that the availability of neurosurgeons to care for the 1.5 million Americans with traumatic brain injuries is increasingly sparse, precipitating a nationwide crisis for neurosurgeon availability in trauma centers.^15^ Similar influences hold true for the relative paucity of other subspecialists, such as orthopedic and trauma surgeons. It is feared that in an effort to maximize staffing efficiency at reduced costs, trauma centers may reduce staff coverage to meet the minimum ACS-COT requirements and trauma center patient demand, with unclear implications to trauma patient’s morbidity and mortality.

The future viability of trauma centers is vulnerable to the escalating cost of care provided to uninsured patients. The transfer of uninsured patients from smaller for-profit hospitals to larger nonprofit hospitals may result in a transfer of financial burden. Previous studies have shown that such transfers are not influenced by insurance status, but rather by injury severity and the presence of multiple injuries.^16^,^17^ However, one three-year study estimates the proportion of uninsured trauma patients seen at an urban Level I trauma center was 37%. In that report, the trauma center lost $37.5 million over three years, mostly attributable to patients without insurance, and Medicare and Medicaid beneficiaries.^18^

Unfortunately, despite the implementation of in-hospital evidence-based guidelines and standardized treatments and best practices to improve the quality of trauma care, substantial variation in patient outcomes occur across trauma centers.^19^,^20^ Therefore, it is important to determine the most critical resources within a hospital that contribute to the treatment of patients with severe injuries. While some research has focused on patient volume in a trauma centers,^21^–^23^ this study examines the proportion of critically ill patients that need the services of a trauma center. To date, no study has focused on the complex relationships between the critically injured patient needing major trauma center services and the hospital resource use, using data from a larger number of trauma centers throughout the U.S. Research in this topic may lead to more efficient trauma resource utilization, and an enhanced ability to meet future trauma care needs. Our primary objective in this study was to examine the how system characteristics impact the proportion of critically injured seen at a trauma center. We hypothesized that both trauma center infrastructure characteristics, patient characteristics and the trauma center staffing levels would be significant predictors in determining the proportion of patients who need care at a trauma center (“trauma center need”). Understanding the important characteristics related to trauma patient admissions to trauma centers may lead to a more efficient use of resources.

## METHODS

This study was a retrospective secondary data analysis using data from the 2009 National Trauma Data Bank (NTDB) Research Data Set. The 2009 NTDB contains trauma registry-based data from participating trauma centers across the U.S. These data are consolidated by the ACS-COT and are a voluntary, convenience sample of trauma center activity in the U.S. For this study, each hospital is the unit of analysis. There is no gold standard for the identification of patients who need the specialized services of a trauma center. To determine trauma center need, (or the portion of critically injured persons who required the services of a trauma center, the outcome variable), this study used a slightly modified version of the definition established by Lerner.^24^ We defined trauma center need as a patient having one or more of the following characteristics: 1) death prior to hospital discharge, 2) ICU admission from the emergency department (ED); or 3) admission to an operating room from the ED for non-orthopedic surgical procedures. This definition was applied to each patient record and a proportion of trauma center need was calculated for each facility. We assigned each hospital a percentage based on the proportion of critically injured patients that required the services of a trauma center, divided by the total number of patients treated. That percentage of critically injured patients in a trauma center served as the outcome variable of the statistical models.

The independent variables that influenced trauma need were hospital infrastructure variables (total beds [adult and pediatric], ICU Beds [adult and pediatric], hospital profit type, trauma designation Level), and we obtained hospital staffing levels (number of neurosurgeons, trauma surgeons, and orthopedic surgeons) from the facility table within the NTDB. When ACS trauma center verification level information was not available, we substituted the state designation for the trauma center level. Other independent variables included staffing levels by specialty (number of trauma surgeons, neurosurgeons and orthopedic surgeons), infrastructure variables such as bed counts and ICU capacity, trauma center designation and hospital type. Variables used to define hospital characteristics, such as the percent of patients transferred and percent of patients insured, were calculated for each trauma center from all of the patients treated within each specific trauma center, and we merged those data with the trauma center.

We omitted patients with isolated orthopedic injuries from the sample, based on the procedural codes in patient records. The International Classification of Diseases, version 9, clinical modification (ICD-9-CM) procedure code range (76–83) was used to identify patients not requiring the services of a trauma center.^25^ Consistent with Lerner’s definition of trauma center need, critically injured patients who were admitted to the operating room from the ED only for operations on the musculoskeletal system were defined as not having a need for a trauma center.^24^ However, if there was a single orthopedic procedure that involved an amputation following a traumatic injury to the limb, we considered those records as trauma related and included them in the trauma care need definition. Unlike Lerner et al., for non-orthopedic cases, we did not limit our analysis to patients receiving surgery within 24 hours of ED arrival. Analysis was performed with SAS, version 9.3.^26^ We used a generalized linear model (GLM) procedure, which is an enhanced multiple regression procedure, to determine how much variance each independent variable contributed to trauma center need. Three models were run to evaluate factors that contribute to trauma center need for: 1) all trauma centers in the NTDB, 2) Level I and II trauma centers, and 3) Level III and IV trauma centers. We performed a tolerance test with a cutoff Level of 0.4 for all three models, which revealed no multi-colinearity issues. Descriptive statistics and parameter statistics are reported.

## RESULTS

A total of 443 trauma centers were used in the statistical models. These trauma centers had 716,898 admissions as reflected in the trauma registries. When looking at all trauma centers, the average proportion of patients meeting one or more inclusion criteria for trauma center need was 31.7% (Table 1). The average percentage of trauma care need in Level I and II trauma centers was 35.3% and 18.6% in Level III and IV trauma centers. The average number of total beds within a Level I and II trauma center was 460 beds and 200 beds within a Level III and IV trauma center. ICU beds were more abundant in Level I and II trauma centers (mean=30.7) than in Level III and IVs (mean =12.7).

A slight majority of the sampled trauma centers were ACS verified or state designated as Level II (n=182 or 41%), followed by Level I (n=165, 37%), Level III (n=74, 17%), and Level IV (n=22, 5%). For all three models (Table 1), there was a higher proportion of nonprofit trauma centers (84%–93%) compared to for profit trauma centers. The percent of insured patients was consistent across the three models at 78–79%. The percent of transfers to a Level I and II trauma centers was almost twice as large when compared to Levels IIIs and IVs (24.1% and 12.2%, respectively).

Staffing with the trauma center groupings was also different. There were almost three times as many neurosurgeons at Level I and II trauma centers (mean=4.9) than at Level III and IV trauma centers (mean=1.8). Similarly, orthopedic surgeons in Level I and II trauma centers also outnumbered other orthopedic surgeons in Level III and IV trauma centers (mean=10.2, mean=6.9, respectively). There were almost twice as many trauma surgeons at Level I and II trauma centers (mean=6.1) than at Level III and IVs (mean=3.6). The overall generalized linear model predicting the proportion of acute trauma center need within all trauma centers (Levels I–IV) was significant (*R^2^* = 0.29, *f*=19.65, *p* <0.001). While the overall model was significant, only certain factors contributed to explaining the proportion of trauma need at a hospital (Table 2). For example, when looking at all trauma centers, specific infrastructure variables such as total beds, ICU beds, and hospital profit type were not significant. We found that a higher percentage of inter-facility transfers (t=2.75, p=0.0061) and a lower percentage of insured patients (t=−2.11, p=0.0356) were associated with higher trauma center need. This model also showed that trauma center designation level category (Level I and IIs combined) was significantly associated with trauma care need (t=8.0, p< 0.0001). Across the entire trauma center care spectrum, staffing resources analyzed (orthopedics, neurosurgeons and trauma surgeons) did not significantly contribute to the predictability of trauma care need.

When examining portion of critically injured patients requiring the services of a trauma center within Level I and II trauma centers only, three independent variables significantly contributed to estimating trauma care need. Nonprofit trauma centers (t = −2.78, p <0.0058) and trauma centers that had a higher percentage of transfers (t = 3.16, p <0.0017), meaning they received more patients transferred from other hospitals, were associated with a higher portion of trauma center need. These transfers were associated with greater need for trauma care. Finally, trauma centers with a lower percentage of insured patients were associated with greater need for trauma center care. (t = −2.95, p <0.0034).

The generalized linear model looking at Level III and IV trauma centers revealed that a larger proportion of patients requiring the resources of trauma center was associated with a larger number of trauma surgeons (t = 2.02, p <0.0464). This effect with not found when looking at orthopedic surgeons or neurosurgeons. This effect was not present when only looking at Level I and II trauma centers.

## DISCUSSION

In examining the data for Level I and II trauma centers, a significant predictor of trauma center need was nonprofit hospital status. This finding could be a result of a higher number of nonprofit trauma centers in the dataset, and across the U.S. Also, the characteristics of the patients in the catchment area and referral pattern of hospitals treating patients at risk for serious injuries are thought to be features more frequently associated with nonprofit trauma centers.

The percentage of transfers that a trauma center received was a clear factor in estimating the proportion of critically injured patients within a trauma center. This effect was significant in two of the three models and was marginally significant (p=0.096) when looking at Level III and IV trauma centers only. Patients are typically transferred to higher level trauma centers because those facilities offer a higher level of care through staffing, resources, and equipment that is not available at lower level trauma centers and non-trauma hospitals.

When we individually examined staffing and infrastructure, those factors were not typically found to be significantly associated with trauma center need. Thus, trauma center infrastructure and staffing levels at Level I and II trauma centers did not influence the proportion of severe trauma seen. Only within Level III and IV trauma centers was the number of trauma surgeons predictive of trauma care need.

### Staffing

Several factors may account for unexpected staffing findings at Level I and II trauma centers. First, the NTDB does not define the term “core trauma surgeon,” which is used in the NTDB dictionary to identify trauma surgeons, and it is unclear how trauma centers interpret the term in their reporting. Therefore, this term may account for all general, trauma, and critical care surgeons who may provide care at a particular facility. Complicating this definition is the fact that the ACS only requires general surgeons who meet specific criteria (board certified, clinical involvement, national and regional involvement, and education) to staff trauma centers. Support for this explanation of the findings can be found in a study where the performance of general emergency surgeons was compared to the trauma surgeons and there was no difference in mortality.^27^ Secondly, this finding may be a reflection of the internal staffing practices and internal call rotations. Third, there is evidence that the use of “closed” ICU environments, with specialized critical care (intensivist) physicians managing patients, has had a positive impact on patient outcomes and resource utilization^28^,^29^ and may impact trauma surgeon staffing patterns at facilities with a high volume of trauma. In a survey of 295 Level I and II trauma centers, Nathans, et al. found that 61% of Level I facilities and 22% of Level II facilities provided an intensivist model of critical care delivery.^30^

The number of neurosurgeons at a trauma center was not associated with trauma center need. This was a surprising finding. Intuitively, neurosurgeon availability should closely track with trauma center need because of the expertise necessary to treat traumatic brain injury (TBI), set forth in the established Brain Trauma Foundation guidelines.^31^ Successful adherence to these guidelines requires immediate and continuous expertise in the management of TBI, most readily available in a Level I or II trauma center as a part of a comprehensive inclusive trauma system, where resources and staffing are an important part of the trauma center verification process.^32^ This finding may be likely reflective of the limited number of neurosurgeons in the U.S. There are only 3,500 neurosurgeons to provide care for a population of 300 million, and closures of trauma centers have been reported to be due in part to a lack of neurosurgical coverage.^33^ Neurosurgeons also often provide care at multiple hospitals, perhaps further limiting the total numbers of neurosurgeons reported by any given facility. Future research using this definition of trauma care need might be useful in determining how staffing levels for neurosurgeons predict trauma care needs for traumatic brain injury.

### Hospital Characteristics

The lower the percentage of insured patients within a Level I or II trauma center, the higher the proportion of patients requiring trauma center resources. This may be reflective of the “safety net” role that many of our nation’s trauma centers play, caring for a large number of uninsured patients. Because Level I and II trauma centers are primarily located in urban settings, these facilities receive patients where violence is widespread. It has been shown that up to 40% of the injuries treated were repeat victims of violence and most of these patients were uninsured (58%).^34^ Additionally, inappropriate transfers to trauma centers may be impacting this finding as well. In a study of patients with orthopedic injuries transferred to Level I trauma centers, Thakur, et al. reported that 52% were inappropriate transfers, and that the majority of inappropriate transfers were uninsured.^35^ This transfer effect was not found in Level III or IV trauma centers. Hospitals receiving a larger percentage of transferred patients also have higher proportions of patients requiring critical trauma resources. This is not surprising, as severely injured patients are typically transferred to higher levels of care for specialty expertise and for the management of complex injuries.^36^

Total beds and total number of ICU beds were not associated with trauma center need. One explanation of these findings is that the number of ICUs and the total number of beds within a hospital may not be solely dedicated to trauma care and are used for the treatment of non-injured patients. Another explanation is that the treatment reputation of a trauma centers may benefit from a “halo effect,”^37^ as documented by trauma centers performing abdominal aortic aneurysm repairs in non-injured patients. The authors suggested that a trauma center has the ability to immediately mobilize both vascular and general surgeons for the patient requiring urgent operative intervention. Thus, the beneficial effects of a trauma center might extend beyond caring for the critically injured and might also enhance the trauma center reputation, which in turn may produce more transfers of critically injured patients to a specific trauma center.

Field triage decisions made by emergency medical services (EMS) personnel certainly impact the destination hospital for injured patients transported by ambulance and the critically ill are more frequently transported by ambulance. However, many injured patients are not transported by EMS resources. In 2010, there were approximately 130 million visits to EDs in the U.S. of which 16.3% were transported by ambulance.^38^ In 2003,there were 40.2 million ED visits for injury and only 6.5 million EMS transports for injury.^39^ A higher percentage of critically injured patients (i.e., those who require the services of a trauma center) are likely to arrive by ambulance, but many are transported to EDs by the public or other modes, such as the police,^40^ and their hospital destination may not be influenced by field triage guidelines, local resources, or personnel of a formal EMS system. Also, the states and localities are free to modify the field triage guidelines or not follow them at all. In efforts to ensure the critically injured are transported to a trauma center, the ACS, in 1990, indicated that an acceptable rate of over triage is 50%. Good adherence to the field triage guidelines can reduce over triage. Thus, adherence to these guidelines, and the management of overtriage via ambulance transports was beyond the scope of this study.

In summary, trauma center need appears to be related to trauma center designation level, hospital type (profit vs. nonprofit), transfers, insurance status, and with the number of trauma surgeons and neurosurgeons in Level III and IV trauma centers. Staffing, bed count or ICU capacity had no significant influence on the proportion of trauma center need. Insurance status of patients and patients who are transferred may be two factors driving the need for trauma services. The results highlight the need for hospital administrators to have a thorough understanding of the patients they serve. The results also suggest that patient characteristics must be considered when deploying a trauma system within a state or region. Inclusive trauma systems help reimburse providers for the un-compensated care of uninsured trauma patients and help distribute trauma cost throughout the system. This study helps shed light on how uninsured patients disproportionately contribute to trauma center need and the importance of the accuracy of inter-facility transfer decisions in determining the proportion of patients admitted to a higher level of care.

## LIMITATIONS

The primary limitation of this study is its retrospective design, where trauma registry data with a limited set of hospital-level variables were available for analysis. Although each analysis model produced significant overall results, the portion of variance explained by the independent variables was low (29% or lower), despite the incompleteness of the NTDB. Thus, the data may not capture most of the factors that influence the portion of trauma need within a facility. Additional factors that influence trauma center need within a hospital may be geography, multiple trauma centers competing for patients and differences in field triage practices. These real world complexities would be difficult to capture in any study. This study also included mostly nonprofit Level I and II trauma centers, which may have impacted the accuracy of the volume of transfer and uninsured patients. Furthermore, using either the ACS-verified and state trauma center designations as a way to define the trauma designation level in this study introduces inconsistencies in defining a trauma center. Defining the trauma center need at the patient level involved a complex composite approach, which is not universally recognized by trauma researchers. As noted in previous trauma literature, trauma science would benefit from an established definition of “true trauma;” i.e. severe injuries requiring the resources of a trauma center, or an acceptable gold standard. We used a slightly modified version of trauma need, as established by previous researchers,^24^ but this definition has not been validated. Finally, we were limited to the variables provided by the NTDB, providing few infrastructures, patient characteristic, and staffing variables for analysis. Information about patient populations served by a trauma center and the percentage electing to be transported to a particular center were unavailable.

## CONCLUSION

Trauma center need is more highly associated with patient characteristics (insurance or transfer status) than hospital facility characteristics. We identified that critically injured patients are often uninsured patients treated in non-profit trauma centers or transferred from lower levels of care. These results can have implications for the role of a trauma system in trauma center reimbursement for uncompensated care. This study may provide insights for hospital administrators and clinicians when planning the construction of new trauma centers or expansion/reduction in current center resources, or when adapting to changes in patient population catchment areas.

## Figures and Tables

**Table 1:** Trauma center characteristics within the national trauma databank.

	Trauma centers			Level I and II trauma centers			Level III and IV trauma centers		
	
	number of centers	Mean	Standard deviation	Number of centers	Mean	Standard deviation	Number of centers	Mean	Standard deviation
Trauma center need*	443	31.7	13.9	347	35.3	12.2	96	18.6	11.8
Infrastructure									
Total beds	443	403.8	231.0	347	460.3	221.7	96	199.7	124.1
Intensive care unit beds	443	26.8	22.7	347	30.7	24.0	96	12.7	7.2
Trauma designation level									
Level I	165 (37%)	-	-	165 (48%)	-	-	-	-	-
Level II	182 (41%)	-	-	182 (52%)	-	-	-	-	-
Level III	74 (17%)	-	-	-	-	-	74 (77%)	-	-
Level IV	22 (5%)	-	-	-	-	-	22 (23%)	-	-
Hospital type									
For profit	40 (9%)	-	-	25 (7%)	-	-	15 (16%)	-	-
Non profit	403 (91%)	-	-	322 (93%)	-	-	81 (84%)	-	-
Patient characteristics									
Percent insured	443	78.7	13.7	347	78.6	14.0	96	78.8	12.5
Percent transferred in	443	21.5	18.5	347	24.1	17.8	96	12.2	18.0
Staffing resources									
Number of neurosurgeons	443	4.2	3.0	347	4.9	2.8	96	1.8	2.6
Number of orthopedic surgeons	443	9.4	6.9	347	10.2	6.9	96	6.9	6.4
Number of trauma surgeons	443	5.6	2.7	347	6.1	2.4	96	3.6	2.5

* Dependent variable of the model. Inference statistics for trauma center need are a summary of the entire model.

**Table 2:** Hospital characteristics associated with truama center need.

	All trauma centers			Level I and II trauma centers			Level III and IV trauma centers		
	
	f-value	t-value	Probability	f-value	t-value	Probablity	f-value	t-value	Probability
ΔTrauma center need	19.65	-	<0.001**	3.79	-	<0.001**	2.99	-	<0.01**
Infrastructure									
Total beds	-	−0.27	0.789	-	−0.59	0.557	-	1.34	0.182
Intensive care unit beds	-	1.62	0.107	-	1.8	0.073	-	0.74	0.461
Trauma designation level									
Level I and II	-	8	<0.0001**	-	-	-	-	-	-
Level III and IV	-	-	-	-	-	-	-	-	-
Hospital type									
Non profit vs. for profit	-	−1.68	0.094	-	−2.78	0.0058**	-	0.64	0.526
Patient characteristics									
Percent transferred in	-	2.75	0.0061**	-	3.16	0.0017**	-	1.68	0.096
Percent insured	-	−2.11	0.0356**	-	−2.95	0.0034**	-	−0.03	0.979
Staffing resources									
Number of neurosurgeons	-	0.87	0.383	-	0.05	0.962	-	1.15	0.253
Number of orthopedic surgeons	-	0.71	0.477	-	1.1	0.272	-	−0.84	0.401
Number of trauma surgeons	-	1.71	0.087	-	0	0.999	-	2.02	0.0464**

Δ Dependent variable of the models. Inference statistics for Trauma Center Need are a summary of the entire model(s).

** Significant at the 0.05 probability level or lower.
